# Variation and genetic basis of mineral content in potato tubers and prospects for genomic selection

**DOI:** 10.3389/fpls.2023.1301297

**Published:** 2023-12-22

**Authors:** Jeewan Pandey, Sanjeev Gautam, Douglas C. Scheuring, Jeffrey W. Koym, M. Isabel Vales

**Affiliations:** ^1^ Department of Horticultural Sciences, Texas A&M University, College Station, TX, United States; ^2^ Texas A&M AgriLife Research and Extension Center, Lubbock, TX, United States

**Keywords:** *Solanum tuberosum*, macronutrients, micronutrients, GWAS, GS

## Abstract

Malnutrition is a major public health concern in many parts of the world. Among other nutrients, minerals are necessary in the human diet. Potato tubers are a good source of minerals; they contribute 18% of the recommended dietary allowance of potassium; 6% of copper, phosphorus, and magnesium; and 2% of calcium and zinc. Increased public interest in improving the nutritional value of foods has prompted the evaluation of mineral content in tubers of advanced genotypes from the Texas A&M Potato Breeding Program and the investigation of the genetics underlying mineral composition in tubers. The objectives of this study were to i) assess phenotypic variation for mineral content in tubers of advanced potato genotypes, ii) identify genomic regions associated with tuber mineral content, and iii) obtain genomic-estimated breeding values. A panel of 214 advanced potato genotypes and reference varieties was phenotyped in three field environments in Texas for the content of 12 minerals in tubers and genotyped using the Infinium Illumina 22K V3 single nucleotide polymorphism (SNP) Array. There was significant variation between potato genotypes for all minerals evaluated except iron. As a market group, red-skinned potatoes had the highest amount of minerals, whereas russets had the lowest mineral content. Reds had significantly higher P, K, S, and Zn than russets and significantly higher P and Mg than chippers. Russets had significantly higher Ca, Mg, and Na than chippers. However, the chippers had significantly higher K than the russets. A genome-wide association study for mineral content using GWASpoly identified three quantitative trait loci (QTL) associated with potassium and manganese content on chromosome 5 and two QTL associated with zinc content on chromosome 7. The loci identified will contribute to a better understanding of the genetic basis of mineral content in potatoes. Genomic-estimated breeding values for mineral macro and micronutrients in tubers obtained with StageWise will guide the selection of parents and the advancement of genotypes in the breeding program to increase mineral content in potato tubers.

## 1 Introduction

Nutrient-rich food is essential to achieve and maintain good health and well-being. The human body needs vitamins and minerals to function properly (Akram et al., 2020). Despite the significant growth in agricultural production, mineral malnutrition is common in developing and developed nations (Miller and Welch, 2013). Low levels of minerals in the human diet can result in severe disorders, developmental impairments in children, reduced resistance to infections, and poor health (Tulchinsky, 2010).

Potato tubers contain important nutrients such as carbohydrates, high-quality protein, vitamins, minerals, and dietary fiber (McGill et al., 2013). Potato varieties with increased levels of minerals have great potential to alleviate mineral malnutrition. Major minerals in potato tubers include nitrogen (N), phosphorus (P), potassium (K), calcium (Ca), magnesium (Mg), sulfur (S), and minor and trace minerals include sodium (Na), zinc (Zn), iron (Fe), copper (Cu), manganese (Mn), boron (B), iodine (I), silicon (Si), and bromine (Br) (Navarre et al., 2009; Beals, 2019). Potatoes have more K per gram than bananas, oranges, and mushrooms (McGill et al., 2013). It is well acknowledged that maintaining a healthy diet includes getting the right amount of minerals. Potato provides 18% of the recommended dietary allowance of K; 6% of Fe, P, and Mg; and 2% of Ca and Zn (Navarre et al., 2009). The bioavailability of Fe in potatoes is higher than that of many other iron-rich vegetables due to the extremely low levels of antinutrients (phytates) that prevent Fe absorption; in addition, the high levels of vitamin C in potatoes have been shown to improve Fe absorption (Beals, 2019).

Potato is one of the most widely consumed and economically important crops worldwide. In the U.S., the per capita consumption of potatoes exceeds 45 kg per year (Davis et al., 2023). In areas where meat is scarce or avoided (vegetarian groups), anemia and malnutrition are common (Aspuru et al., 2011). Potato tubers constitute a significant dietary source of minerals and thus, even small improvements to the chemical composition of potatoes to make them healthier, more nutritious, and more flavorful could significantly impact human health and well-being (Burgos et al., 2020). Enhancing nutrients, bioactive compounds, and quality traits in tubers have value-added potential for the processing and fresh market potato industries. Information about potatoes’ distinctive nutrients and bioactive compounds could help increase consumer acceptance and selling potential of existing potatoes and new releases (Bamberg and Greenway, 2019).

Enhancing micronutrient nutrition through supplements, food fortification, and the development of crop cultivars with improved nutrition have all been successful (Haynes et al., 2012). Developing micronutrient-enhanced plants via breeding is economically feasible and a more viable long-term solution than supplementing or fortifying through industrial procedures (Hansson et al., 2018). HarvestPlus, one component of the Consortium of International Agricultural Research Centers (CGIAR) research program on agriculture for nutrition and health, and its collaborators have demonstrated the effectiveness of plant breeding in alleviating micronutrient deficiencies since 2003 (Bouis and Saltzman, 2017). In potatoes, the mineral content of tubers is influenced by genetic and environmental factors (Karan, 2023), making breeding more challenging. For the past couple of decades, the International Potato Center (CIP) has been working on potato mineral biofortification to raise the amount of iron and zinc (Burgos et al., 2020), but other potato breeding programs have placed more emphasis over the past few decades on increasing yield potential, processing quality, and improving disease/pest resistances, typically without paying much attention to the mineral composition of tubers. Several studies have revealed significant genetic variation for mineral accumulation in potato tubers, pointing to the possibility of breeding for high mineral levels (Andre et al., 2007; Brown et al., 2010; Brown et al., 2011; Brown et al., 2012; Haynes et al., 2012; Brown et al., 2013; Brown et al., 2014; Subramanian et al., 2017; Navarre et al., 2019). There are no studies linking the mineral content of tubers with specific market groups (reds, russets, and chippers). This knowledge gap emphasizes the need to explore the mineral content of various potato market groups represented by advanced genotypes in a breeding program.

Given the significance of mineral nutrition in staple foods, it is important to identify genes or molecular markers that could be used for the indirect selection of nutrition and quality traits. Innovative manufacturing companies are keen to use minerals-rich varieties to improve nutrition, and therefore high mineral content in food crops has emerged as a crucial selection attribute for breeders (Bouis and Saltzman, 2017). Improved knowledge about the genetic basis of mineral content in tubers (genes, quantitative trait loci - QTL, heritability) will be useful to guide breeding to increase the mineral content in potato tubers. Few studies have targeted the genetic basis of mineral accumulation in potato tubers. Subramanian (2012) conducted QTL analysis for tuber mineral concentrations using a tetraploid mapping population (12601abl x Stirling) and reported that a significant number of genes/QTL were involved in the accumulation of mineral elements in potato tubers. Genome-wide association studies (GWAS) have been used to dissect the genetic basis of traits in potatoes (Rosyara et al., 2016; Sharma et al., 2018). GWAS has been conducted in potatoes for protein content (Klaassen et al., 2019), scab resistance (Kaiser et al., 2020), root and stolon traits (Yousaf et al., 2021), tuber traits (Pandey et al., 2022), tuber bruising (Angelin-Bonnet et al., 2023), and tuber-bound free amino acids (Pandey et al., 2023b), etc. However, there has been no prior instance of GWAS for mineral content in potato tubers based on our knowledge.

Genomic prediction utilizes the genomic information of individuals and relatives to predict their phenotypic performance. In potatoes, studies have demonstrated the potential of genomic selection for chipping quality (Sverrisdóttir et al., 2018; Pandey et al., 2023a), disease resistance (Enciso-Rodriguez et al., 2018), fry color (Byrne et al., 2020), that could represent a faster and cost-effective alternative to traditional phenotypic evaluations in the case of using marker-based selection or re-enforce/improve breeding values estimates if both phenotypic and genotypic data are used. Genomic-estimated breeding values can be used to select parents and advance genotypes in the breeding pipeline. Applying genomic predictions would accelerate genetic gains in developing nutrient-enhanced potato varieties.

Increased public interest in improving the nutritional value of foods has triggered the evaluation of mineral contents in advanced genotypes from the Texas A&M Breeding Program and the investigation of the genetics underlying mineral content in tubers. This study hypothesizes that there is significant variation in the mineral content of tubers of the advanced potato genotypes and that the differences are due, in great part, to genetic factors; thus selecting clones based on genomic-estimated breeding values for high mineral content should result in healthier and more nutritious potato tubers. Therefore, the objectives of this research were to i) assess phenotypic variation for mineral content in tubers of advanced potato genotypes, ii) identify genomic regions associated with tuber mineral content, and iii) obtain genomic-estimated breeding values.

## 2 Materials and methods

### 2.1 Plant materials and experimental design

The study was performed using 214 tetraploid potato genotypes. The panel comprised 31 chippers, 62 russets, 32 yellows, 68 reds, and 21 purples (Pandey et al., 2021). It included reference varieties [Russet Norkotah, Atlantic, Russet Burbank, White LaSoda, and a Yukon Gold strain (TXYG79)] and advanced genotypes from the Texas A&M Potato Breeding Program. In 2019, genotypes were evaluated in Dalhart (35°58′N, 102°44′W), Texas, and in the year 2020, the genotypes were evaluated in Dalhart and Springlake (34°6′N, 102°19′W), Texas (**Supplementary Table 1**) in 12-hill plots in a randomized complete block design with two replications. The trials were planted, harvested, and fertilized following practices used by local commercial growers (**Supplementary Table 1**). The Springlake trial was planted in March and harvested in July, whereas the Dalhart trials were planted in May and harvested in September (**Supplementary Table 1**). Other production practices, including spacing, irrigation, weed, and pest management are available in the annual reports of the Texas A&M Potato Breeding Program for both 2019 and 2020, accessible at https://potato.tamu.edu/reports/.

### 2.2 Mineral content assessment

Three random tubers (from the 113.4-170.1 g grading category) per plot were diced into small cubes measuring 4 mm on each side and mixed very well. Approximately 15 g of freshly diced samples were placed into 50 mL tubes. Samples were kept in a -80 C freezer for a few days before freeze-drying. Freeze-drying was done at a collector temperature of -50 C for 120 h at 22 Pa. using a Labconco FreeZone 6-L Freeze-dry System (Labconco, Kansas City, MO, USA. To homogenize the dried tuber samples, ceramic grinding cylinders (0.95 cm x 2.22 cm) with angle-cut ends (SPEX SamplePrep, Metuchen, NJ, USA) were used. This homogenization was performed in 50 mL centrifuge tubes using the SPEX Sample Prep 1600 MiniG tissue homogenizer (SPEX SamplePrep, Metuchen, NJ, USA) for two minutes at a speed of 1,500 strokes per minute. Powdered potato samples were used to determine the mineral content at the laboratory (Soil, Water, and Forage Testing Laboratory, Texas A&M AgriLife Extension). Total nitrogen was determined by high-temperature combustion Kjeldahl procedure and expressed in percent (Nelson and Sommers, 1973), and minerals (P, K, Ca, Mg, S, Na, Zn, Fe, Cu, Mn, and B) were determined by inductively coupled plasma-mass spectrometry (ICP-MS) analysis of a nitric acid digest (Havlin and Soltanpour, 1980). Macrominerals were expressed in terms of mg/g and microminerals were expressed in terms of μg/g on a tuber dry weight (DW) basis.

### 2.3 Genotyping and data preparation

Genotypic data for 214 tetraploid potato genotypes were obtained from an earlier study (Pandey et al., 2021) using the Illumina Infinium 22K V3 Potato SNP Array (Illumina Inc., San Diego, CA, United States). Genotype clustering based on the intensities of SNPs was done using GenomeStudio (Illumina Inc., San Diego, CA, United States) after downloading the raw data. The molecular marker dataset was filtered to retain SNPs with a 90% call rate and a minor allele frequency of at least 0.05 (Pandey et al., 2021). After filtering, 10,106 SNP markers were selected for subsequent analysis.

### 2.4 Statistical analyses

Best linear unbiased estimates (BLUEs), minimums, and maximums were obtained for the tuber mineral content of each potato genotype using the software package META-R (Alvarado et al., 2020). Distributions for the mineral content were created, and normality tests were carried out using the Shapiro-Wilk W-Test in JMP Pro 16 statistical software by SAS Institute, Cary, NC, USA. The minerals were grouped using a hierarchical clustering analysis, using Euclidean distance metric and Ward’s minimal variance method in JMP Pro 16. To assess the influence of locations, years, environments (considered as location-year combination: Dalhart 2019, Dalhart 2020, and Springlake 2020), genotypes (comprising 214 genotypes), and the mineral content of raw potato tubers, a mixed model analysis of variance was conducted in JMP Pro 16. Genotypes were treated as fixed effects, while environments, replications within environments, and interactions were considered random. Likewise, an analysis of variance was employed to determine the effect of locations (specifically Dalhart and Springlake field trials conducted in 2020) on the traits. In this instance, both genotypes and locations were regarded as fixed effects. The data from two years (2019, 2020) at one location (Dalhart) were used to assess the influence of years on the evaluated traits. Pair-wise correlation coefficients between minerals for each environment were determined using a multivariate analysis using JMP Pro 16. Also, correlations between minerals and yield-related traits (Pandey et al., 2021) evaluated in Texas (Dalhart in 2019 and 2020, and Springlake in 2020) were calculated. Also, principal component analysis (PCA) was done using the function prcomp in the stats package based on a correlation matrix and plotted using the function ggbiplot from the ggplot2 package in R (Wickham, 2016). Visualization of the PCA plot was done using the factoextra package in R (https://github.com/kassambara/factoextra).

### 2.5 Genome-wide association analysis

Marker-trait association analysis was performed using the dataset for tuber mineral content and 10,106 SNPs using the GWASpoly Version 2 https://github.com/jendelman/GWASpoly) package in R (Rosyara et al., 2016). To take population structure into consideration, the leave-one-chromosome-out (LOCO) method was employed. Both dominant and additive genetic models were evaluated for each trait. A Bonferroni test was used to determine the LOD threshold for each trait, which corresponds to a 5% false-positive rate across the genome. Manhattan plots were produced with the use of the GWASpoly software. The percentage of phenotypic variance at each QTL peak that can be attributed to significant SNPs was calculated using GWASpoly. To find possible candidate genes, contextual sequences of SNPs near the peak of QTL were used in BLAST searches of DM1-3 pseudomolecules (Version 4.03) in the SpudDB database (http://solanaceae.plantbiology.msu.edu/).

### 2.6 Genomic selection

Genetic variance partitioning and genome-wide prediction (genomic-estimated breeding values, GEBVs) were obtained using allele dosage information using the StageWise R package (https://github.com/jendelman/StageWise). Standard errors and the best linear unbiased predictors (BLUP) were calculated using standard methods. For a generic random vector u, BLUP $\left[\text{u}\right]=\hat{\text{u}}=\text{CPy}$ , where C=cov[u,y] , $\text{P}={\text{V}}^{-1}-{\text{V}}^{-1}\text{X}{\left({\text{X}}^{\prime }{\text{V}}^{-1}\text{X}\right)}^{-1}{\text{X}}^{\prime }{\text{V}}^{-1}$ , X is the incidence matrix for fixed effects, and V is the variance-covariance matrix of the response variable (Endelman, 2023; Pandey et al., 2023a). The reliability of ${\hat{u}}_{i}$ is represented by the squared correlation with the true value which equals ${\text{r}}_{\text{i}}^{2}=\frac{\text{var}\left[{\hat{\text{u}}}_{\text{i}}\right]}{\text{var}\left[{\text{u}}_{\text{i}}\right]},$ and $\text{var}\left[\hat{\text{u}}\right]=\text{CP}{\text{C}}^{\prime }$ (Endelman, 2023; Pandey et al., 2023a).

### 2.7 Weighted standardized multitrait selection indexes

Standardized multi-trait selection indexes were calculated for mineral content based on the Z values of GEBVs (Pandey et al., 2023a). The weighted multi-trait selection index (Z_WMIS_) for each genotype was obtained by assigning twice the value (2x weight) to Fe and Zn (important for biofortification) and providing equal weights to all other minerals. A Z_WMIS_ >2 was used as a criterion to identify the most promising genotypes based on genomic-estimated breeding values. A Z_WMIS_ >2 indicates that the values are two standard deviations above the mean.

## 3 Results

### 3.1 Phenotypic variation for mineral content in potato

The panel of 214 cultivated tetraploid potato genotypes evaluated in three environments showed significant phenotypic variation for the relative amounts of mineral contents in potato tubers, except for Fe (ns) (**Table 1**). Based on the Shapiro-Wilk W-Test, the frequency distributions of mineral content were normal for N, K, Mg, Fe, Mn, and B, while the distributions for P, Ca, Na, Zn, Cu, and S showed an approximately normal distribution (**Supplementary Figure 1**). The range of macromineral content varied from 2.96 to 4.79 mg/g for P, 20.39 to 32.46 mg/g for K, 0.19 to 0.79 mg/g for Ca, 0.81 to 1.44 mg/g for Mg, and 1.07 to 1.86 mg/g for S content on a dry-weight (DW) basis (**Table 1**). The averages for P, K, Ca, Mg, and S content over all 214 genotypes were 3.69, 25.09, 0.44, 1.14, and 1.42 mg/g DW, respectively. Across the diversity panel, K was the most abundant mineral. Tubers of the genotype ATX85404-8W had the highest K (32.46 mg/g DW), followed by Tacna (31.89 mg/g DW) (**Supplementary Table 2**). The range of micromineral content varied from 102.50 to 468.46 μg/g DW for Na, 13.22 to 27.93 μg/g DW for Zn, 18.71-27.96 μg/g DW for Fe, 2.16 to 6.10 μg/g DW for Cu, 3.85 to 8.92 μg/g DW for Mn and 1.89 to 4.98 μg/g DW for B. The averages for Na, Zn, Fe, Cu, Mn, and B content over all 214 genotypes were 276.38 μg/g, 19.66 μg/g, 22.92 μg/g, 3.82 μg/g, 6.15 μg/g, and 3.38 μg/g DW, respectively. B was the least abundant (**Table 1**). The genotype TX14611-1R had the highest concentration of Zn (27.93 μg/g DW), (**Supplementary Table 2**). Broad-sense heritability ranged from 6% (Fe) to 74% (K and Mg) (**Table 1**). The significant phenotypic variation and moderate to high heritability for most minerals (notable exception of Fe with low heritability) indicate that it should be feasible to investigate the underlying genetic factors that contribute to their expression and to make progress when selecting for high mineral content.

**Table 1:** Range, average (in dry weight basis), and broad sense heritability (H^2^) of macro and micro mineral content of 214 potato genotypes evaluated in three environments in Texas: Dalhart 2019, 2020, and Springlake 2020.

Macro Minerals	Units	Min	Max	Avg.	LSD	H^2^ (%)	Micro Minerals	Units	Min	Max	Avg.	LSD	H^2^ (%)
**N**	%	1.32	2.33	1.83	0.23	53.0	**Na**	μg/g	102.50	468.46	276.38	105.50	53.0
**P**	mg/g	2.96	4.79	3.69	0.38	68.0	**Zn**	μg/g	13.22	27.93	19.66	3.82	67.0
**K**	mg/g	20.39	32.46	25.09	2.67	74.0	**Fe**	μg/g	18.71	27.96	22.92	ns	6.0
**Ca**	mg/g	0.19	0.79	0.44	0.18	55.0	**Cu**	μg/g	2.16	6.10	3.82	0.86	68.0
**Mg**	mg/g	0.81	1.44	1.14	0.12	74.0	**Mn**	μg/g	3.85	8.92	6.15	1.20	66.0
**S**	mg/g	1.07	1.86	1.42	0.17	72.0	**B**	μg/g	1.89	4.98	3.38	0.72	34.0

LSD, Least significant difference; N, nitrogen; P, phosphorus; K, potassium; Ca, calcium; Mg, magnesium; S, sulfur; Na, sodium; Zn, zinc; Fe, iron; Cu, copper; Mn, manganese; B, boron.

For N, Mg, S, Na, Zn, Mn, and B significant interactions between genotypes and environments were found (**Supplementary Table 3**). Significant differences were detected between genotypes evaluated for all minerals evaluated in Dalhart in 2019 and 2020, and Springlake in 2020, except for the Fe content (**Supplementary Table 3**). Analysis of variance from the two Texas locations (Dalhart and Springlake) in 2020 indicated that for all minerals except for Fe and B, significant interactions between genotypes and location were found (**Supplementary Table 3**). However, most mineral contents were not affected by growing location, except P, Na, and Zn (**Supplementary Table 3**). The P content was significantly higher (36.2% higher) in Dalhart (3.99 mg/g) than in Springlake (2.93 mg/g). The Zn concentration was significantly higher (8.7%) in Dalhart (20.68 μg/g) than in Springlake (19.02 μg/g). However, the Na level was significantly higher (347.8%) in Springlake (509.46 μg/g) than in Dalhart (113.77 μg/g). Analysis of variance using data from two years (2019, 2020, Dalhart location) revealed significant interaction between genotype and year for Ca, Mg, S, Na, Zn, Mn, and B but no significant differences between years (**Supplementary Table 3**). Significant variation was observed for tuber mineral content between potato market groups (reds, russets, purples, yellows, and chippers) for seven (P, K, Ca, Mg, S, Na, and Zn) out of the 12 minerals evaluated (**Table 2**). As a group, red-skinned potatoes had the highest amount of minerals, whereas russets had the lowest mineral content. Reds had significantly higher P, K, S, and Zn than russets and significantly higher P and Mg than chippers. Russets had significantly higher Ca, Mg, and Na than chippers. However, the chippers had significantly higher K than the russets.

**Table 2:** Average values by market groups for mineral content of 214 potato genotypes evaluated in three environments in Texas (Dalhart in 2019 and 2020, and Springlake in 2020).

Market groups	N	P	K	Ca	Mg	S	Na	Zn	Fe	Cu	Mn	B
	(%)	(mg/g)	(mg/g)	(mg/g)	(mg/g)	(mg/g)	(μg/g)	(μg/g)	(μg/g)	(μg/g)	(μg/g)	(μg/g)
Reds	1.91A	3.83A	26.08A	0.38AB	1.15A	1.49A	266.39AB	21.39A	23.43A	3.77A	6.50A	3.74A
Russets	1.73A	3.56B	23.39B	0.55A	1.16A	1.36B	342.97A	17.78B	23.60A	3.69A	6.04A	3.31A
Purples	1.84A	3.70AB	26.08A	0.382AB	1.13AB	1.42AB	227.53B	20.33A	22.71A	3.63A	6.15A	2.83A
Yellows	1.79A	3.69AB	24.84AB	0.45AB	1.11AB	1.38B	234.20B	19.66AB	20.86A	3.96A	6.13A	3.48A
Chippers	1.89A	3.63B	26.01A	0.31B	1.08B	1.39AB	221.66B	19.66AB	22.63A	4.01A	5.76A	3.02A

**Figure 1:**
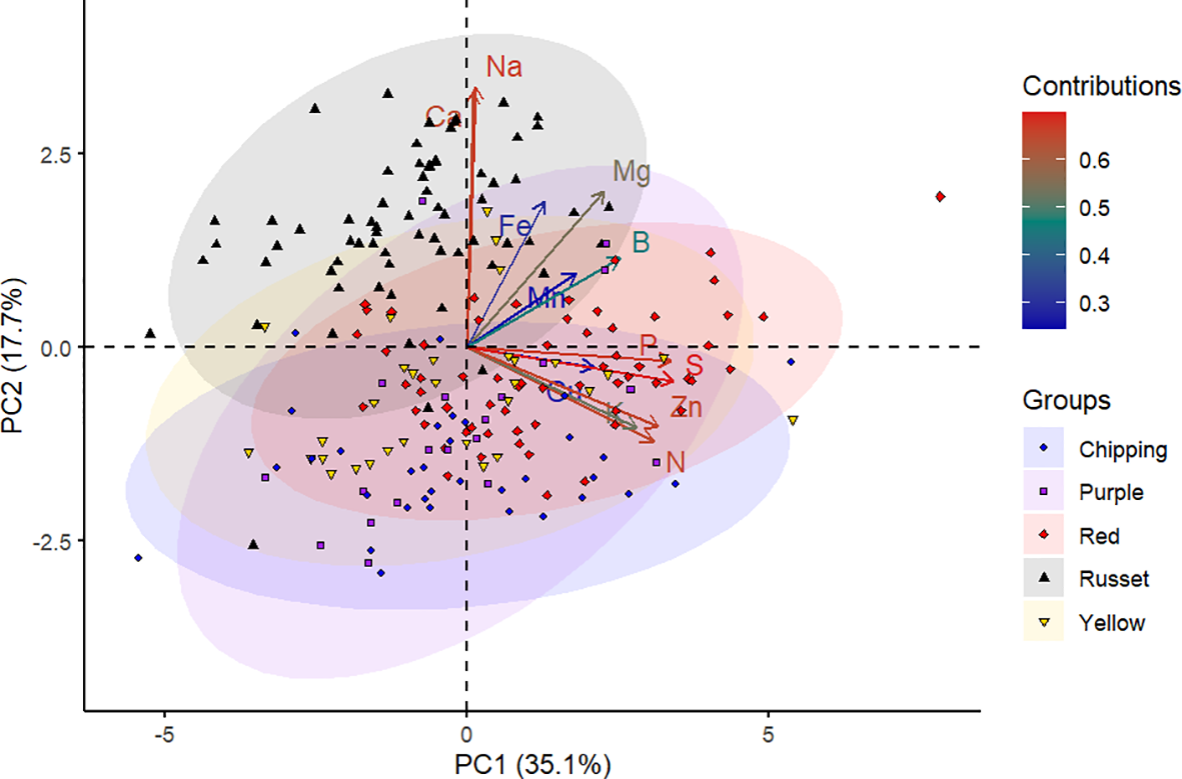
Principal component analysis of 214 potato genotypes belonging to various market groups (chipping, purple, red, russet, and yellow) based on tuber mineral content. Each shape on the plot represents an individual genotype, and the shape colors represent the market groups. Each shape represents the projection of an individual potato genotype in the PC1 and PC2 axes. The ellipses represent 95% confidence intervals around the centroid of each data cluster. The contributions (%) of minerals to PC1 and PC2 from low to high are indicated by blue to red color gradients, respectively.

The panel of genotypes was subjected to PCA based on mineral content and separation between market groups. The first two principal components accounted for 52.8% of the total variance in the data, wherein the first principal component (PC1) explained 35.1% and the second principal component (PC2) explained 17.7% of this variance (**Figure 1**). The S, P, Na, Zn, N, Ca, Mg, and K contributed the most to the PC1 and PC2 as observed by the intense red color in the contributions scale (**Figure 1**, **Supplementary Figure 2**).

**Figure 2:**
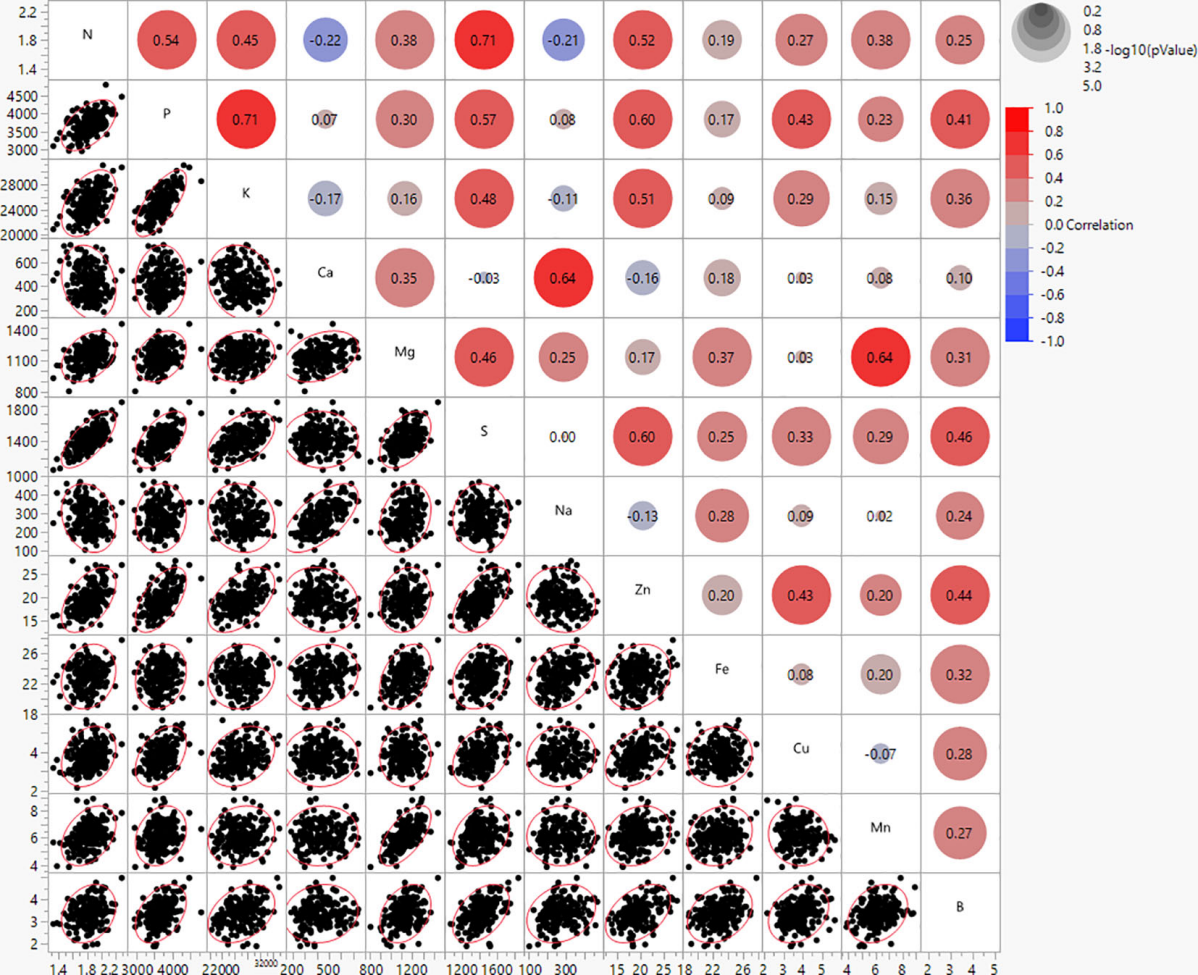
Bivariate scatter plots (bottom of the diagonal) and the value of the correlation (top of the diagonal) for mineral content of 214 potato genotypes evaluated in three environments in Texas: Dalhart 2019, 2020, and Springlake 2020.

The mineral contents in potato genotypes were found to have strong to weak correlations (**Figure 2**). N and S showed the highest positive correlations (r = 0.71), and N and Ca were found to have weak negative correlations (r = -0.22) (**Figure 2**). The correlations between minerals and average tuber weight per plant (yield per plant) were not significant, however, the correlations with yield components (tuber weight/tuber, tuber number/plant, and tuber shape) were weak and/or moderate (**Supplementary Table 4**). Ca and Na showed positive correlations with average weight per tuber (r = 0.43 and 0.33, respectively) and tuber shape (r = 0.56 and 0.48, respectively) indicating that large, long tubers had more Ca and Na than small, rounded tubers. However, Ca and Na showed negative correlations with the average tuber number per plant (r = -0.36 and -0.34, respectively), thus more Ca and Na were present if plants produced fewer tubers. K and Zn showed negative correlations with average weight per tuber (r = -0.39 and -0.35, respectively) and tuber shape (r = -0.45 and -0.39, respectively), but showed positive correlations with average tuber number per plant (r = 0.27 and 0.29, respectively), thus more K and Zn was present in smaller, rounder tubers, and when plants produced a higher number of tubers (**Supplementary Table 4**).

**Figure 3:**
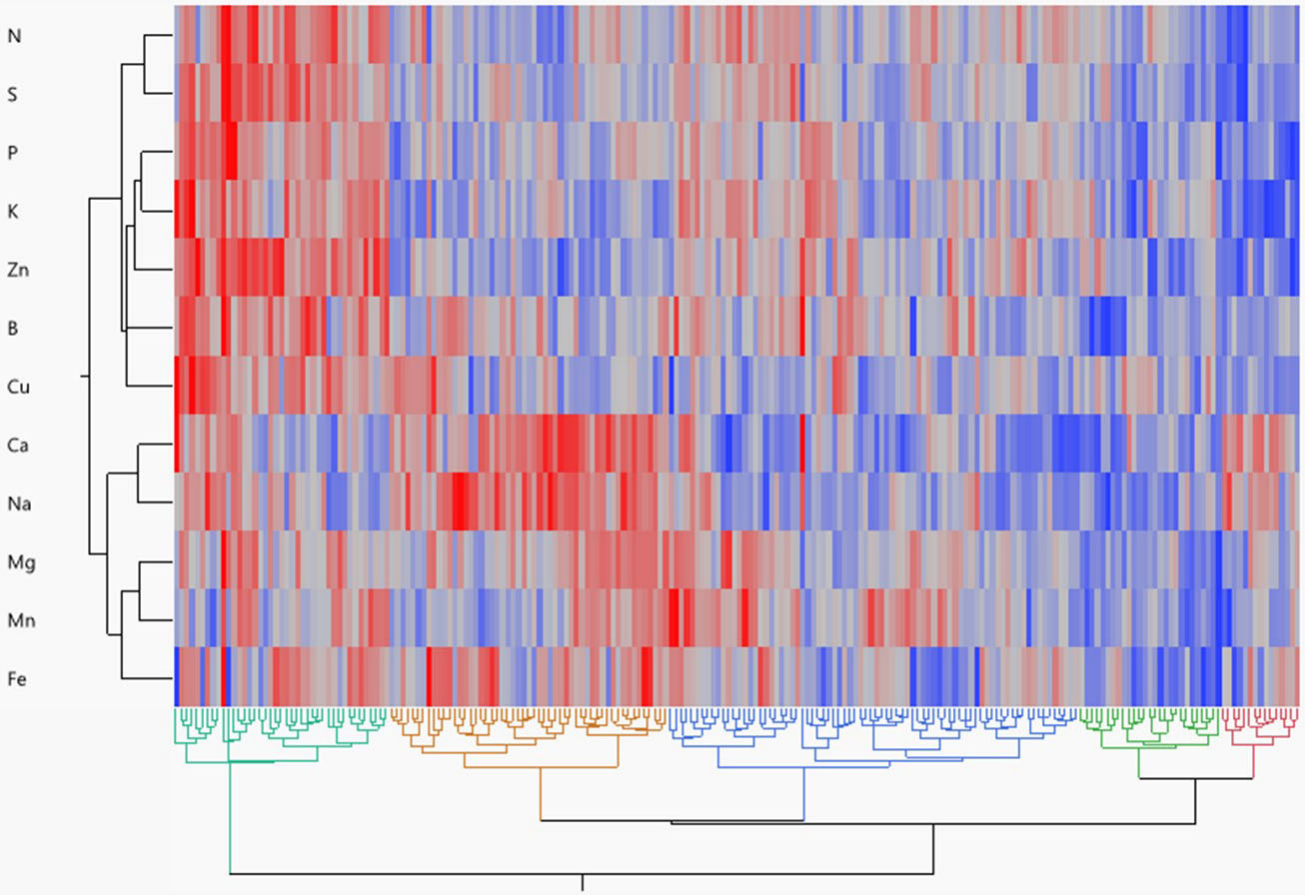
Hierarchical cluster two-way analysis (Ward method) of tuber mineral content across 214 tetraploid potato genotypes. Each genotype is visualized as a single column. Each row represents an individual mineral. Red denotes higher mineral content, whereas blue denotes lower mineral content.

Hierarchical cluster analysis reflected the metabolic relationship between mineral content in potatoes as well as the genetic relationship between potato genotypes (**Figure 3**). Five genotypic groups were obtained from a dendrogram based on Ward’s method. Group 1 included 15 genotypes (mainly russets) that are high in Na and Ca but low in all other minerals. Group 2 included 27 genotypes (mainly chippers and yellows) having low amounts of all minerals. Group 3 comprised 78 genotypes (mainly reds with yellow flesh) and had the highest amount of Mn and the lowest amount of Ca and Na. Group 4 encompassed 53 genotypes (mainly russets) having high Ca, Na, Mg, and Fe. Group 5 included 41 genotypes (mainly reds without colorful flesh) having the highest amounts of all minerals.

### 3.2 Genome-wide association studies for mineral content in potato tubers

**Figure 4:**
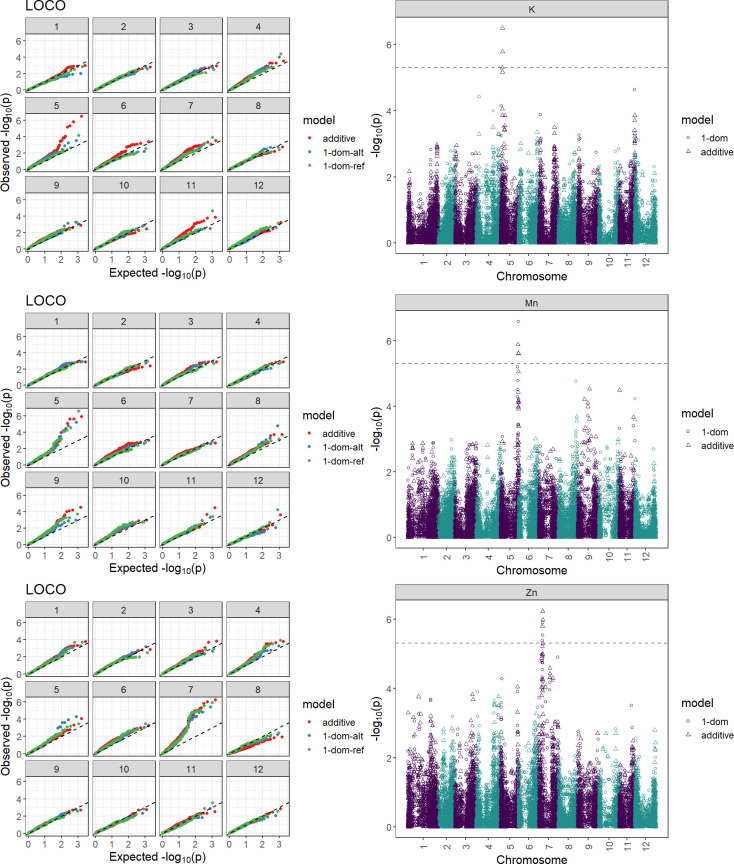
Quantile-quantile plot and Manhattan plots displaying marker-trait associations for the tuber minerals K, Mn, and Zn by GWASpoly using the additive and dominant model. Two hundred and fourteen potato clones were genotyped (Infinium Illumina 22K V3 SNP Array) and evaluated for tuber mineral content in three environments (Dalhart, Texas in 2019 and 2020, and Springlake, Texas in 2020). The horizontal axes of the Manhattan plots indicate the chromosome number and the position of each SNP. The vertical axes indicate the negative logarithm of the P-value for each SNP. Each dot signifies an SNP. The broken line indicates the Bonferroni threshold level of 0.05.

To discover chromosomal regions that contribute to the phenotypic variation, GWAS was employed with additive and simplex dominant models of inheritance for the 12 mineral contents. The inflation of the -log10(p) was examined using quantile-quantile (QQ) plots of the observed vs. expected values under the null hypothesis (**Figure 4**). Significant marker-trait associations were identified for K, Mn, and Zn (**Figure 4**, **Table 3**). Three QTL associated with K and Mn content on chromosome 5 and QTL associated with Zn content on chromosome 7 were identified. For other minerals, no significant associations were found.

**Table 3:** Significant marker-trait associations identified for the mineral content of potatoes obtained from the evaluation of 214 potato genotypes in three field experiments in Dalhart, Texas in 2019 and 2020, and Springlake, Texas in 2020.

Mineral	Model	Threshold	SNP at QTL peak	Chr.	Peak Position (bp)	Score [-log10(p)]	R^2^ (%)
K	additive	5.31	PotVar0079038	5	4488420	6.59	2.2
Mn	additive	5.31	PotVar0128091	5	49730536	6.29	2.0
Mn	1-dom-alt	4.97	PotVar0128091	5	49730536	5.30	2.0
Mn	1-dom-ref	5.09	PotVar0034950	5	50863328	6.68	2.2
Zn	additive	5.31	solcap_snp_c2_6617	7	10836607	6.29	2.0
Zn	1-dom-ref	5.09	PotVar0037204	7	55079806	5.53	2.0

The genes found in each peak SNP’s 5 Mbp flanking genomic region were taken from the reference genome of potatoes (**Supplementary Table 5**). When looking into candidate genes, choosing a 5 Mbp interval is standard practice. The potential function of candidate genes was assessed using UniProt (www.uniprot.org) and DM version 4.03 gene annotations. The roles of several of the genes were unknown (**Supplementary Table 5**). However, our study revealed a few putative genes that are closely connected to the primary metabolic pathways. On chromosome 5, the potassium transporter gene (PGSC0003DMG400004113) is a candidate within the K QTL. Several gene families, such as Zn ion binding protein (PGSC0003DMG402015931), zinc knuckle family protein (PGSC0003DMG400040608 and PGSC0003DMG400039749), and zinc finger protein (PGSC0003DMG400038007), are among the potential genes for Zn QTL on chromosome 7 (**Supplementary Table 5**). Given the degree of linkage disequilibrium in potatoes, it cannot be ruled out that genes regulating mineral content could be located megabases away from important SNPs.

### 3.3 Genomic selection

#### 3.3.1 Genomic-estimated breeding values for traits

**Figure 5:**
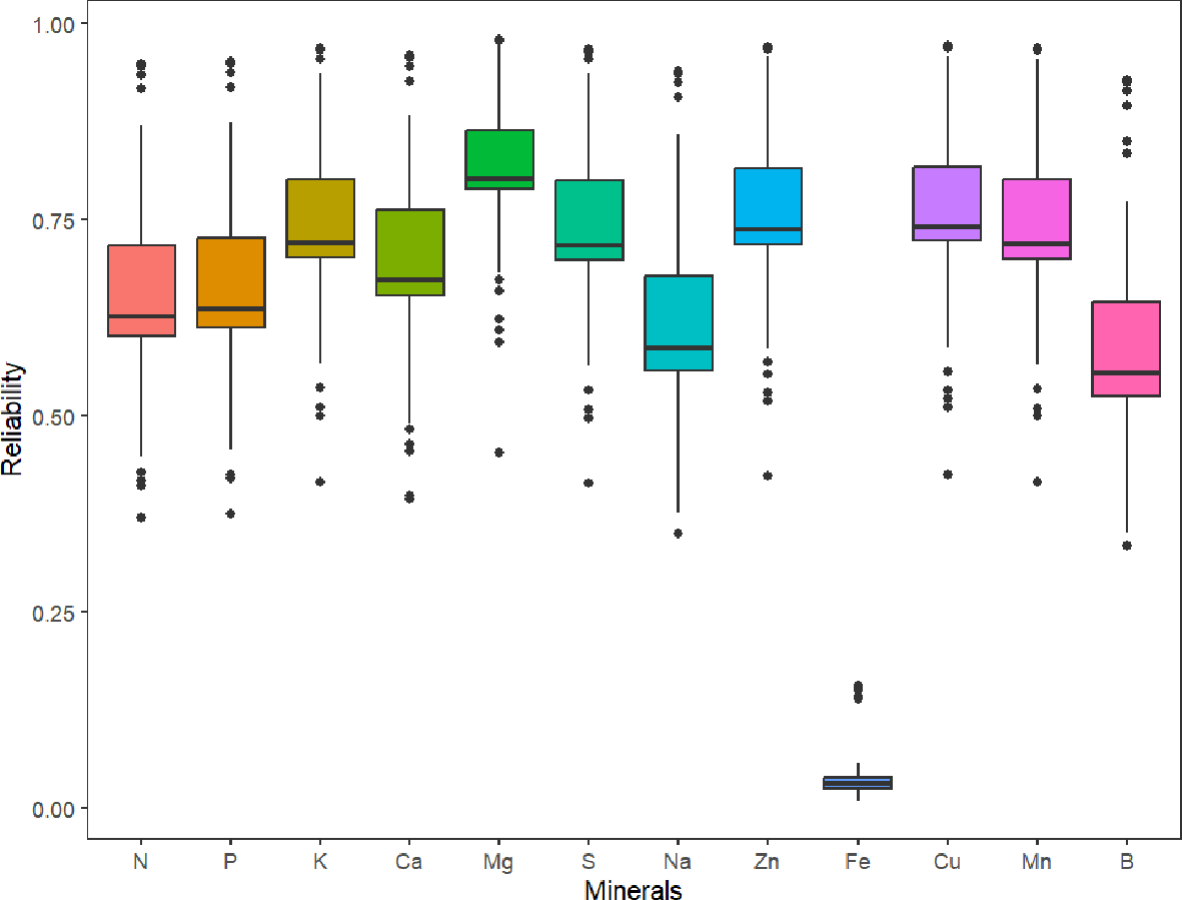
Box plot of the predicted reliabilities (the squared correlation between the true and predicted values) for minerals content in 214 potato genotypes evaluated in three environments (Dalhart, Texas in 2019 and 2020, and Springlake, Texas in 2020).

Parental selection and the advancement of superior genotypes in breeding programs can be guided by genomic estimated breeding values. The predicted reliabilities for all minerals (which is the squared correlation between the true and predicted values), with the exception of Fe, are generally higher than 0.5, according to the box plot of the predicted reliabilities (**Figure 5**).

#### 3.3.2 Weighted standardized multitrait selection indexes

Genotypes with the highest GEBVs for tuber mineral content based on weighted normalized multitrait selection indexes (Z_WMIS_-score >2) were: NDTX050169-1R, COTX94218-1R, COTX00104-6R, TX10437-9Pyspl/Y, BTX2332-1R, and ATTX01178-1R (**Supplementary Table 6**) meaning that they were the best candidates as parents to increase the mineral content of potato tubers. Among them, NDTX050169-1R, a genotype with red skin and white flesh, and a high number of small tubers, had the highest mineral content of all genotypes tested and a very high Z_WMIS_ (4.2). Most of the other genotypes with Z_WMIS_-score >2 also had red skin and white flesh (except TX10437-9Pyspl/Y, a purple-splashed skin genotype with yellow flesh).

## 4 Discussion

Knowledge about the phenotypic variation available for tuber mineral content of potato genotypes belonging to various market groups, along with the identification of genomic regions underlying the traits are important considerations in determining plant breeding strategies to improve nutrient content. In addition, the use of GEBVs based on weighted multiple traits has emerged as one of the best recommendations to ensure progress in developing varieties with enhanced tuber mineral composition. One of the most effective strategies to address global nutrient deficiencies is to increase mineral accumulation (biofortification) in crops. Biofortification approaches for nutrient enrichment of crops encompass agronomic, conventional breeding, and transgenic/biotechnological methods (Dhaliwal et al., 2022).

Identifying cultivars with varying mineral concentrations is beneficial for breeding programs. The panel used in this study represents a collection of advanced genotypes from the Texas A&M University Potato Breeding Program and reference varieties belonging to various market groups, making them a valuable resource for studying the genetic variation in mineral content. In this study, the phenotypic distributions for mineral content in potato tubers were normal for most minerals (**Supplementary Figure 1**), implying that the traits are quantitatively inherited. The mineral amounts reported were similar to those published in prior research; the differences in range could be attributed to differences in the potato varieties evaluated, environments, as well as phenotyping methodology. In our study, the phenotypic variation for P and K (**Table 1**) aligns with previous research, such as Subramanian et al. (2017), who reported similar mineral content for P and slightly lower values for K. The K level for breeding lines and established varieties ranged from 19.0 to 25.0 mg/g DW in a study conducted in the Pacific Northwest (Navarre et al., 2019). Additionally, our study’s phenotypic variations for Ca, magnesium Mg, S, Fe, and Zn demonstrated consistency with the ranges reported in other studies (Bamberg et al., 1993; Paget et al., 2014; Subramanian et al., 2017). However, Andre et al. (2007) reported a wide range of Fe content in the potato cultivars native to the Andes of South America from 29.9 to 154.9 μg/g DW.

Most minerals had moderate to high broad-sense heritability (34.0% to 74.0%) across the three Texas environments, except for Fe (6.0%). Moderate to high broad-sense heritability indicates that the phenotype is primarily driven by the genotypic effects and indicates that breeding efforts can make progress through selection (Piepho and Möhring, 2007). Similar moderate to high broad-sense heritabilities for minerals have been discovered in earlier studies (Brown et al., 2010; Brown et al., 2011; Brown et al., 2012; Haynes et al., 2012; Brown et al., 2013; Brown et al., 2014; Paget et al., 2014; Asfaw et al., 2018; Seid et al., 2023). Regarding Fe, the heritability estimates reported were not consistent. The likely reason for obtaining low estimates of broad sense heritability for Fe is that despite observing some variation, there were no significant differences between genotypes for the Fe level in tubers. Paget et al. (2014) and Haynes et al. (2012) reported moderate heritability for Fe (44% and 49% respectively) whereas Brown et al. (2010) reported 0%, 64%, and 73% in the Tri-State, Western Regional Russet, and Western Specialty/Red Trials, respectively. Incorporation of potato genotypes with high Fe (from CIP, for example) in crossing blocks is recommended to enrich Fe content in tubers. Greater breeding challenges will arise in situations with low heritability as there will be less confidence in the selection of superior genotypes (Rebetzke et al., 2002). Low heritability traits often require larger populations and more test environments than traits having high heritabilities for selection and improvement (Merrick et al., 2023).

Furthermore, positive correlations were found in this study between several minerals (**Figure 2**), implying that the selection to increase one mineral will be accompanied by increases in other minerals. In a few instances, the increase in one mineral did not affect the change in others, and moderate significant negative correlations were detected in the following cases: an increase of N resulted in a decrease of Ca and Na. Peña et al. (2015) reported strong positive correlations between Fe and S (0.66), Fe and Zn (0.67), Mg and Mn (0.80), and P and K (0.69). Andre et al. (2007) showed Zn and Fe contents to be weakly correlated (r = 0.35). Our study did not find a significant correlation between Fe and Zn, likely because the phenotypic variation for Fe was low and no significant variation was detected between genotypes for Fe. According to Burgos et al. (2007), the correlation between Fe and Zn may change depending on the location. In our study, no significant correlations were found between tuber yield per plant (complex trait) and tuber mineral content. However, when the yield was dissected, correlations between minerals and yield components (weight/tubers, tuber number/plant, and tuber shape) were significant, yet low to moderate. A positive correlation between average tuber weight with Ca and Na indicates that larger tubers have, in general, higher Ca and Na levels. Potatoes from the russet market group typically have heavier tubers. However, the negative correlation of average tuber number per plant with Ca and Na suggests that if plants produced a higher number of tubers per plant, then tubers would have lower Ca and Na levels. Negative correlations between average tuber weight with K and Zn imply that small tubers may contain higher levels of K and Zn. Positive correlations between average tuber number per plant with K and Zn indicate that plants that produce more tubers may have an increased K and Zn in tubers. These findings imply that when altering yield components there is likely a tradeoff of minerals and it could be very challenging to increase at once all minerals in tubers. The theory that an increase in production can result in decreased levels of minerals is supported by some studies (Garvin et al., 2006; Davis, 2009), although it is not universally accepted. Regardless of the “diluting” phenomena, interactions between minerals in the soil and fertilizer can change the mineral concentration in tubers (White et al., 2009). Our findings suggested that Ca and Na are predominant in elongated, large tubers, and lower in round, small tubers. Likewise, K and Zn are predominant in round, small tubers and lower in elongated, large tubers.

In our study, significant differences among genotypes were found in all scenarios except for Fe, which suggests that significant genetic variation exists for most of the tuber minerals content in potatoes. Environment (Dalhart 2019, 2020 and Springlake 2020) and years (Dalhart 2019, Dalhart 2020) did not significantly affect the content of minerals in tubers whereas location (Dalhart 2020, Springlake 2020) had a significant effect in P, Na, and Zn (**Supplementary Table 3**). However, significant interactions between genotypes and environments were identified for N, Mg, Na, Zn, Mn, S, and B (**Supplementary Table 3**). Likewise, significant interactions between genotypes and location were identified for all minerals except for Fe and B (**Supplementary Table 3**). Also, significant interactions between genotypes and year were identified for Ca, Mg, Na, Zn, Mn, S, and B (**Supplementary Table 3**). In agricultural studies, this result is not unusual. Different factors can affect crops’ mineral content, including the genotypic effect, however, some minerals are more sensitive to changes in soil composition and conditions than others (Delgado et al., 2001; Lombardo et al., 2013). The uptake and accumulation of minerals in the tubers may differ depending on the soil types, soil mineral concentrations, and climatic conditions (White et al., 2009; Wekesa et al., 2014). Fertilization, irrigation, and washing of water-soluble soil minerals due to excessive rain can also play a major role in the final mineral composition of tubers.

Some of the minerals varied significantly amongst potato market groups (**Table 2**). P and S were significantly higher in reds than in russets and chippers, whereas russets had significantly higher Na than purples, yellows, and chippers, and significantly lower K and Zn than reds. The Ca and Mg levels were significantly lower in chipping genotypes than in russets. Previous literature has not reported the mineral content of potato genotypes separated by market groups such as reds, russets, and chipping genotypes. For nutritional analysis and agricultural practices, it is essential to comprehend the differences in mineral content among different potato market groups. This study offers insightful information about the composition of various market groups for enhancing the mineral content. On average, the red-skin white flesh market group had the highest content of minerals in tubers, whereas the russet group had the lowest levels. However, there was variation within each group.

The tuber mineral data was displayed in a two-dimensional biplot using PCA (**Figure 1**). The biplot demonstrated how the genotypes and the mineral elements were related to the principal components. Genotypes in the PCA biplot (**Figure 1**) were dispersed among four quadrants, indicating a substantial genetic variation among them. The length of the vectors indicated how much influence they had on that PC. In this study, it is evident that the vectors corresponding to tuber minerals S, P, Na, Zn, N, Ca, Mg, and K are mainly responsible for the separation of genotypes. According to Mwadzingeni et al. (2016), a small angle between variables indicates a positive correlation, a large one implies a negative correlation, and a 90° angle suggests no correlation. In this study, Na and Ca, Mn and B, and P and S were found to have narrow angles, showing a positive interaction between them. From the PCA, Ca and Na are the characteristic mineral elements in the russet genotypes (**Figure 1**). Additional studies with mineral samples of various market groups need to be investigated to confirm this point.

Different genotype clustering patterns were identified by cluster analysis utilizing a neighbor-joining algorithm and Ward’s method (**Figure 3**). The 214 genotypes were divided into five separate clusters based on 12 minerals, demonstrating the genotypes showing significant genetic differences in terms of measured traits. The members of the fifth cluster (mainly red skin white flesh genotypes) showed the highest concentrations for most of the minerals and thus could serve as a gene pool for potato breeding programs aiming at increasing the concentration of minerals in tubers. Parents from genetically distinct groups can be used to create breeding populations with desirable nutritional traits (Souza and Sorrells, 1991; Gerrano et al., 2019).

Genome-wide association studies are effective at locating genomic areas that are responsible for regulating quantitative traits affected by numerous loci (Korte and Farlow, 2013; Desaint et al., 2023). The genetic basis of mineral content in potatoes is still poorly understood (Haynes et al., 2012; Singh et al., 2021) and this study represents one of the few reports on genome-wide association studies for mineral content in tetraploid potatoes. This study investigated the potential and limitations of genome-wide association studies in tetraploid potato panels using genotypic data gathered with the 22K V3 Potato array. GWASpoly (Rosyara et al., 2016) takes population structure and relatedness into account to filter out erroneous relationships. As a result of the GWAS in this study, two QTL associated with Zn content on chromosome 7 and three QTL associated with K and Mn content on chromosome 5 were identified explaining around 2% each of the phenotypic variance (**Table 3**). Some QTL were also detected in previous studies for mineral traits using QTL mapping. For example, Mengist et al. (2018) reported five QTL for tuber Zn content on chromosomes 1, 3, 5, and 6 explaining 5.0-38.0% of the phenotypic variance. Subramanian (2012) reported QTL associated with Zn on seven different chromosomes and the one on chromosome 7 was exhibited over three years, explaining about 6.6 to 9.6% of the total phenotypic variance. Although our study did not find QTL for Ca, Zorrilla et al. (2021) utilizing the mapping population from the cross of Atlantic and Superior, identified five QTL on chromosomes 1, 3, 4, 5, 7, and 8 to explain tuber Ca variation. Subramanian (2012) reported QTL associated with K on chromosome 5 which explained 5.1 and 6.1% of the phenotypic variance. Also, QTLs for tuber Mn concentrations were detected on eight chromosomes by Subramanian (2012) and the QTL on chromosomes 2, 5, and 12 were consistent over the three years. Certain traits are suitable for GWAS but some experience greater drawbacks from the limitations of GWAS. Vos et al. (2022) recommended utilizing the combination of GWAS and bi-parental mapping populations to prevent inaccurate data interpretation. The panel used in this study was not intentionally selected to show extensive variation for all minerals present in tubers and thus has limitations in detecting the genetic basis (QTL, genes) behind certain minerals. Nevertheless, advanced selection panels like this developed based on selection for other traits like yield, disease resistance, abiotic stress tolerance, etc. could be exploited for the identification tuber mineral QTL, however, using specific populations that maximize genetic variation for the traits of interests and mapping populations involving contrasting parents for the traits of interest (in this case tuber mineral content) should be considered to maximize the detection of tuber mineral QTL.

The annotated potato reference genome was used to identify potential candidate genes in the discovered QTL. The candidate genes within the identified QTL for Zn on chromosome 7 include several gene families such as zinc ion binding protein (PGSC0003DMG402015931), zinc knuckle family protein (PGSC0003DMG400040608 and PGSC0003DMG400039749), and zinc finger protein (PGSC0003DMG400038007). Zinc ion binding proteins and zinc finger proteins coordinate zinc ions, bind nucleic acids, and regulate a wide range of cellular processes in plants, including growth, development, and responses to biotic and abiotic stimuli. Qin et al. (2019) reported that the plant homeodomain (a type of zinc finger domain) genes were unequally scattered on the chromosomes of potatoes and chromosome 7 contained the largest number of plant homeodomain genes. Likewise, the candidate gene within the identified QTL for K on chromosome 5 includes a potassium transporter (PGSC0003DMG400004113). Potassium transporter genes play critical roles in the uptake, distribution, and signaling of potassium (Li et al., 2022). The approximate locations of potassium transporter genes on each chromosome were indicated by Li et al. (2022) and it was found that chromosome 5 contained just one gene. Functional analysis of the identified candidate genes would help to determine how they affect the mineral contents in potatoes. The identified QTL may be of considerable utility for effective application in the marker-assisted breeding of minerals in potatoes.

In contrast to GWAS, genomic selection accounts for the effects of multiple genes controlling a trait and allows the selection of superior individuals based on GEBVs (Jannink et al., 2010). When genomic selection is used in the early stages of breeding projects, there are several benefits, including decreased costs associated with assessing phenotypes and quicker development of new cultivars (Slater et al., 2016; Hickey et al., 2017). Software like StageWise, which supports both polyploidy and dominance heterosis, has recently been created for the study of multi-environment, multi-trait datasets for genomic selection in polyploid crops. We believe that this study is the first to document tuber mineral predictions in potatoes using weighted multitrait standardized scores (Z score). Our findings show that different tuber minerals can be predicted across environments with moderate to high reliability except Fe. We found that prediction reliability for the tuber mineral content was severely impacted by heritability which was the reason for low reliability in Fe. For practical breeding purposes, other traits (marketable yield, specific gravity, shape, chip quality, disease/pest resistance, etc.) should be incorporated in the multitrait standardized scores. The list of traits and the weight of each trait will be specific for each market group, regional preferences, and breeders’ perspective. Breeders should keep in mind that high heritability of target traits is required in the training/reference population to achieve good prediction accuracy values in members of the prediction/breeding/candidate population. Variable levels of accuracy values have been reported in plants for different traits, depending on prediction models, breeding scheme, the size of the training population, the relationship between the training and prediction populations, the complexity of the trait, marker densities, and genotyping platforms (Jia and Jannink, 2012). According to Zhang et al. (2017), trait heritability has the strongest correlation with prediction accuracy estimation, even more than training population size and marker density. GEBVs for many traits may be combined into an overall index to employ GEBVs for selection (marker-based selection - if only markers are used, or marker-assisted selection - if markers are used together with phenotypic data to improve the estimation of the breeding value of genotypes). We used the standardized indexes for the tuber mineral contents in this analysis. Breeders could choose genotypes as parents or advance them in the breeding program by obtaining a weighted multi-trait selection index (WMIS) that includes tuber mineral content and several other weighted important traits that can differ depending on the market group and final desired selection criteria.

## 5 Conclusions

Crop nutrient content can be considerably enhanced if it is given priority and includes expanding genetic and genomic resources and fast, efficient, and ideally non-destructive testing technologies. The present study is the first detailed work using an advanced potato panel to identify genomic regions associated with tuber mineral traits using genome-wide association studies and to document the tuber mineral genomic-estimated predictions in potatoes. The results obtained from this study will be useful in developing potato cultivars with improved mineral qualities and alleviating mineral malnutrition in humans.

## Data availability statement

The datasets presented in this study can be found in online repositories. The names of the repository/repositories and accession number(s) can be found in the article/**Supplementary Material**.

## Author contributions

JP: Conceptualization, Data curation, Formal analysis, Investigation, Methodology, Software, Validation, Visualization, Writing – original draft, Writing – review & editing. SG: Writing – review & editing. DS: Writing – review & editing. JK: Writing – review & editing. MV: Conceptualization, Funding acquisition, Investigation, Methodology, Project administration, Supervision, Validation, Writing – review & editing, Resources.
