# Supplementary material for: Variation and genetic basis of mineral content in potato tubers and prospects for genomic selection
Source: Front Plant Sci. 2023 Dec 22;14:1301297. doi: 10.3389/fpls.2023.1301297 (PMC10766833; doi:10.3389/fpls.2023.1301297)
Supplement: Supplementary file 1 [file DataSheet_1.docx]

Supplementary Material


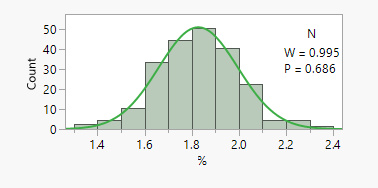

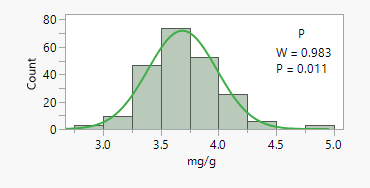


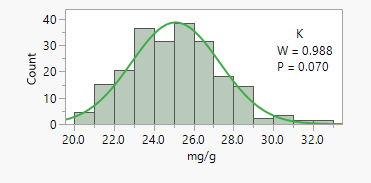

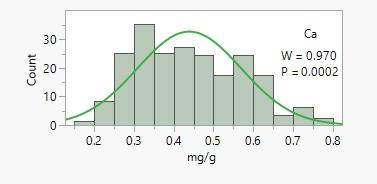


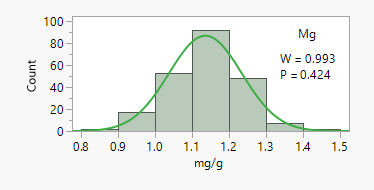

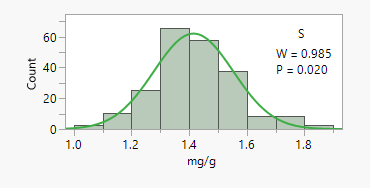


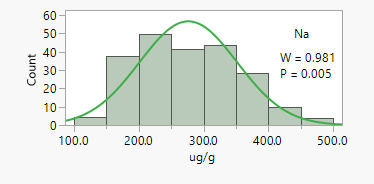

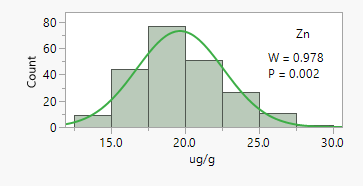


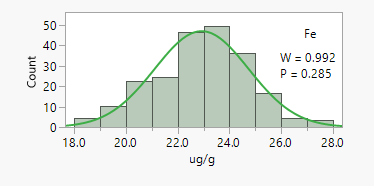

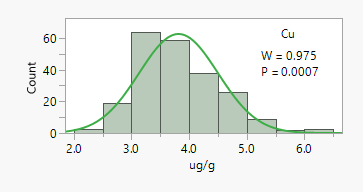


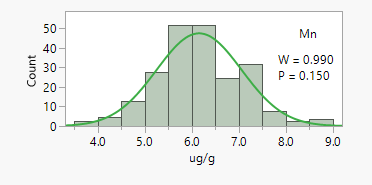

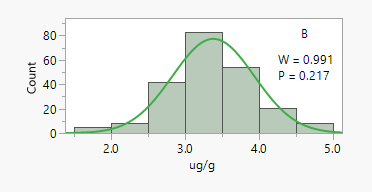


**Supplementary Figure 1: Frequency distributions of mineral content in 214 tetraploid advanced potato genotypes evaluated in three environments of Texas (Dalhart 2019, 2020 and Springlake 2020).** **Both the test statistic (W) and associated p value from a Shapiro–Wilk normality test are shown.**


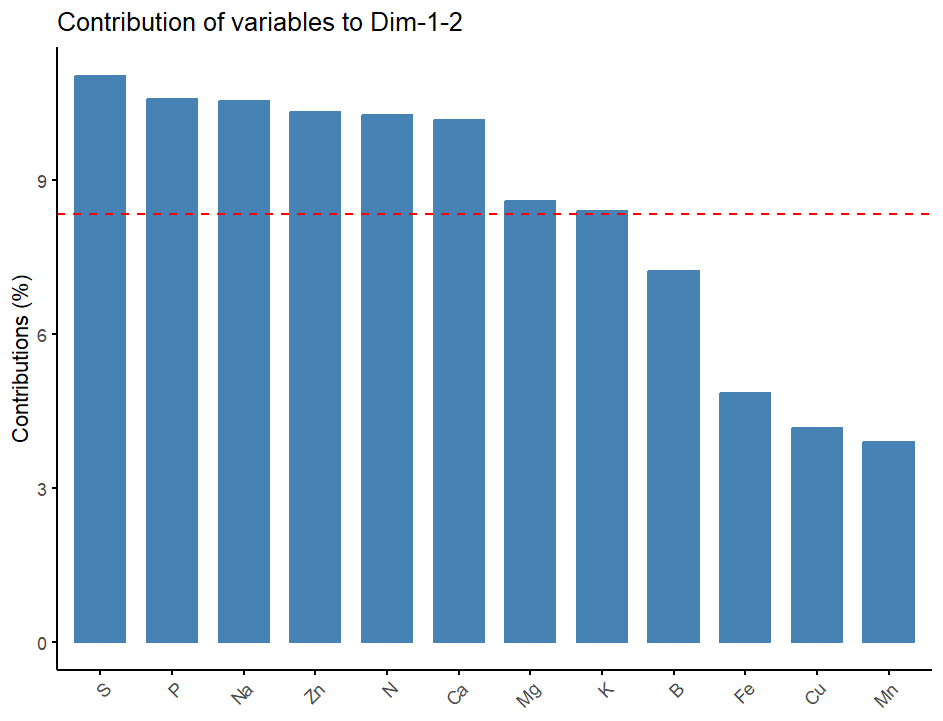


**Supplementary Figure 2:** **Bar plot of** **contributions (%) of mineral contents to dimensions 1 and 2. The red dashed (cutoff) line on the graph indicates the expected average contribution.**
